# Application of Fuzzy Adaptive Impedance Control Based on Backstepping Method for PAM Elbow Exoskeleton in Rehabilitation

**DOI:** 10.3390/polym16243533

**Published:** 2024-12-18

**Authors:** Zhirui Zhao, Xinyu Hou, Dexing Shan, Hongjun Liu, Hongshuai Liu, Lina Hao

**Affiliations:** 1 School of Mechatronics Engineering, Shenyang Aerospace University, Shenyang 110136, China; hou15509824645@163.com (X.H.); shandexing@sau.edu.cn (D.S.); 20052794@sau.edu.cn (H.L.); 2 Suzhou Automotive Research Institute, Tsinghua University, Beijing 100190, China; 1910084@stu.neu.edu.cn; 3 School of Mechanical and Electronic Engineering, Northeastern University, Shenyang 110819, China; haolina@me.neu.edu.cn

**Keywords:** PAM exoskeleton, fuzzy impedance control, backstepping control, rehabilitation

## Abstract

In this study, a fuzzy adaptive impedance control method integrating the backstepping control for the PAM elbow exoskeleton was developed to facilitate robot-assisted rehabilitation tasks. The proposed method uses fuzzy logic to adjust impedance parameters, thereby optimizing user adaptability and reducing interactive torque, which are major limitations of traditional impedance control methods. Furthermore, a repetitive learning algorithm and an adaptive control strategy were incorporated to improve the performance of position accuracy, addressing the time-varying uncertainties and nonlinear disturbances inherent in the exoskeleton. The stability of the proposed controller was tested, and then corresponding simulations and an elbow flexion and extension rehabilitation experiment were performed. The results showed that, with the proposed method, the root mean square of the tracking error was 0.032 rad (i.e., 21.95% less than that of the PID method), and the steady-state interactive torque was 1.917 N·m (i.e., 46.49% less than that of the traditional impedance control). These values exceeded those of the existing methods and supported the potential application of the proposed method for other soft actuators and robots.

## 1. Introduction

The ‘Hardiman’ prototype robot was first reported in the 1960s, and since then, the exoskeleton has been leveraged as a conceptual product to show test power to improve human strength in a master–slave configuration [1,2]. As of the present day, the exoskeleton has undergone significant modifications due to the emergence of high-tech tools that combine human intelligence with robotic strength. It has presented innovative solutions across different applications. On the one hand, military exoskeletons (such as ONYX, XOS, and Guardian XO) have been designed using high-torque drive systems and innovative composite materials [3,4,5,6]. These types of exoskeletons can support the user’s body and improve the loading capacity during lifting tasks as well as promoting the mobility of a soldier on a battlefield.

On the other hand, the field of rehabilitation exoskeletons (used for amyotrophic lateral sclerosis and post-stoke rehabilitation therapy) and advanced orthotics has witnessed significant advancements, including biomechanical designs, lightweight structures, and personalized customization. These devices (such as ANYexo, NEUROExos, and NESM-γ) can restore muscle strength or replace humans’ lost function, improving the quality of life among the elderly and disabled population. These devices not only support individuals with disabilities but also help them lead more self-sufficient lives and perform daily tasks with greater ease. They offer a variety of rehabilitation training exercises, including both passive and active methods [7]. During passive rehabilitation, the exoskeleton or robot moves the patient’s limbs without requiring voluntary effort. In contrast, active rehabilitation requires the patient to actively participate in the movement [7,8,9,10,11]. Taking elbow exoskeletons as an example, these devices are especially vital for individuals recovering from neurological conditions such as stroke. Specifically designed for the elbow joint, they offer precise torque control for both elbow flexion and extension. These exoskeletons are adaptable to the patient’s rehabilitation process, accommodating both passive and active movements. The ultimate goal is to restore the elbow’s flexibility, stability, and strength [12,13].

To achieve precise motion control and provide sufficient power, the above exoskeletons often rely on high-performance servo systems, such as servo motors with high-radio reducers or complicated linkage mechanisms [14]. However, as integrated human–machine devices, actuators used in the exoskeleton have a low power-to-weight ratio and occupy a relatively small volume, which compromises flexibility but optimizes the structure size and inertia of the joints [15]. For this reason, this kind of exoskeleton does not efficiently match the movement characteristics of human joints. Moreover, servo motors, which primarily output continuous torque at the joint and lack back-drivability, cannot accurately replicate the force exertion pattern of the human musculoskeletal system [16]. Collectively, these factors exacerbate user discomfort and pose safety issues during the operation of exoskeletons.

Therefore, it is imperative to incorporate soft actuators and artificial muscles, such as silicone rubber, composite hydrogels, and polymer materials, to ensure comfort and security in the robotic system. Furthermore, some researchers have chosen to use compliant joints to ensure that exoskeletons are both lightweight and durable [17]. Taking the pneumatic artificial muscle (PAM) as an example, this typical soft actuator comprises a sealed rubber hose and high-strength fiber braid, and it exhibits a high power-to-weight ratio. Pneumatic muscles can achieve various types of movements, including contraction, extension, bending, and twisting. Therefore, they can be integrated into exoskeletons [18,19]. Due to their linear force generation principle, flexible architecture, light weight, and capacity to act as an energy carrier, PAMs are extensively applied in a wide range of robotic applications, from soft robotics to the industrial automation. Moreover, the integration of PAMs into exoskeletons, they can allow passive and active upper-limb rehabilitation tasks with a comfortable experience to patients [20,21,22,23].

Despite the above advantages of the PAMs-driven exoskeleton, there are some challenges that limit its application in rehabilitation tasks. One of the major challenges is that the PAM has a typical time-varying nonlinear system caused by the elasticity of the rubber hose and the friction between the rubber hose and the fiber braid under air pressure [24]. Meanwhile, disturbances associated with the human actions and uncertain dynamic models of the system also affect the performance of PAMs [25]. Therefore, strategies for maintaining the accuracy of position control in the PAM-driven exoskeleton need to be explored. In the past, several data-driven control methods (e.g., artificial neural networks, nonlinear PID, and model-free control methods) have been investigated in the PAMs-driven system for position control [26,27,28,29]. These model-free control techniques can overcome the intricate challenges associated with dynamic modeling processes, offering a simpler approach to system regulation. However, tracking the performance is entirely contingent upon the quality of the input and output data [25]. The precision of these data points is crucial, as any inaccuracies or noise may limit the position accuracy, which restricts their effectiveness in applications where high precision is mandatory.

For high-demand tasks such as active rehabilitation, a PAMs-driven exoskeleton should not only control the position accurately but also detect human intent during the operation [30]. A classical method involves the establishment of an interactive model, so-called the traditional impedance model, following a physical human–robot interaction (pHRI) control structure [31]. Within this structure, the interactive force/torque between the exoskeleton and its user, the joint position, and its derivatives (i.e., velocity) are represented using a virtual physical series system with a spring and damper element [32]. The damping and stiffness, referred to as impedance parameters, influence the exoskeleton’s response to external forces and the user’s movements, providing a smooth and safe interaction. This highlights the importance of selecting appropriate parameters during the application of the exoskeleton. The improper selection of the damping parameter can cause the shaking of the exoskeleton under the interactive force/torque, while an incorrect stiffness parameter may pose a safety risk to the user because the exoskeleton can become overly sensitive due to changes in the interactive force/torque. Conversely, setting the damping too high can make the exoskeleton difficult to control, which will constrain human movement and create discomfort for the user [33]. Therefore, impedance control utilizes adaptive algorithms, neural networks, and fuzzy logic algorithms to enhance the ability to fine-tune impedance parameters according to the user’s need. Among the methods mentioned, the fuzzy adaptive impedance control method stands out for its ability to simplify the tuning of parameters and enhance real-time performance. This approach improves the system’s adaptability and robustness, especially when dealing with unpredictable and non-uniform environments. By integrating a fuzzy logic system into impedance control, it enables decision-making based on imprecise inputs, akin to the human interpretation of vague or ambiguous information [34,35,36]. However, a challenge arises in how to balance the effectiveness of adaptive impedance control with the precision of joint position tracking in elbow exoskeletons.

To address the aforementioned challenges, a two-layer architecture control method for the PAM elbow exoskeleton was proposed for elbow flexion and extension rehabilitation tasks in the study. In the lower layer, the controller design was developed based on the backstepping control method to improve the position accuracy of the PAMs-driven exoskeleton. Backstepping control has been successfully used in many nonlinear systems, such as industrial robots, micro-grippers, and continuum manipulators [37,38,39,40]. It is a recursive technique used in nonlinear control systems, which simplifies the stabilization process by systematically reducing the system’s order. To differentiate this study from previous ones, a repetitive learning method was integrated into the lower layer to approximate the periodic distribution of the PAMs-driven exoskeleton, and an adaptive control law was added to correct the tracking errors caused by time-varying uncertainties. In the higher layer, a variable impedance control method based on fuzzy logic was proposed to ensure that the exoskeleton could smoothly follow the movement of the user’s arm. Therefore, this effective approach could be used to adapt the impedance parameters (i.e., damping and stiffness), allowing it to dynamically match the varying impedance of human muscles throughout the range of rehabilitation. The contributions are summarized as follows:

I. Following the principles of fuzzy adaptive control, a two-tier architecture control method was developed for the PAM elbow exoskeleton, designed to adjust impedance parameters for physical interaction during rehabilitation tasks.
II. To enhance position-tracking performance under uncertain time-varying disturbances and modeling errors, a P-type repetitive learning control strategy was integrated with the backstepping control method and an adaptive law.

This paper is organized into five sections as follows: Section 2 provides a brief introduction about the prototype of the PAM elbow exoskeleton. Then, the two-layer architecture control method of the PAM-driven exoskeleton system is discussed. In Section 3, to verify the control effectiveness of the proposed method, we discuss how the PAM elbow exoskeleton prototype was utilized. Then, we describe in detail how several simulations and experiments were conducted to mimic elbow rehabilitation tasks. Lastly, the conclusions drawn are presented in Section 4.

## 2. Materials and Methods

### 2.1. The Design of the PAM Elbow Exoskeleton

**Figure 1:**
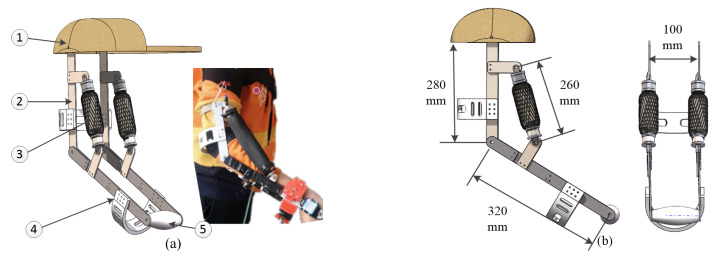
Experimental results: interactive torque ((**a**) prototype of PAM elbow exoskeleton, (1) strap, (2) linkage, (3) PAM, (4) connector, and (5) handle); (**b**) design drawing.

This section introduces the mechanical structure of the PAM elbow exoskeleton. As previously mentioned, two McKibben-type PAMs were used as actuators to achieve flexion and extension movements of the elbow joint. The top and bottom ends of the PAMs were secured within the linkage system, which was constructed from lightweight, high-strength aluminum alloy as shown in Figure 1. To maintain the exoskeleton in a lightweight system, both the handle and connectors were prepared from the PLA material and printed using a 3D printer. Therefore, the whole mechanical structure weighed approximately 1.25 kg (with the following breakdown: the linkage system weighed 260 g, the handle and connections weighed 200 g, the two PAMs weighed 400 g, the strap weighed 300 g, and the other hardware weighed 90 g) [22]. Additionally, the exoskeleton was affixed to the human upper limbs with straps. Using the PAM elbow exoskeleton, users can easily raise their forearms to 90 degrees while lifting 5 kg heavy weights, making it suitable for elbow flexion and extension rehabilitation training. More details are discussed in our previous study [22].

### 2.2. Higher-Layer Control Method: Variable Impedance Control Method Based on Fuzzy Rules

As mentioned in Section 1, the impedance controller is one of the effective methods used in interaction tasks between the exoskeleton and its user. It provides a human–exoskeleton cooperated model with appropriate impedance parameters (i.e., the stiffness and damping of the system), enhancing the admittance ability of the exoskeleton to follow human actions. It also builds a bridge between the human–exoskeleton interactive torque (i.e., $\tau_{h}$) with the motion as follows [41]:

(1) $$\tau_{h}=D\Delta {\dot{q}}_{e}+K\Delta q_{e}$$

where *D* represents the damping of the system; *K* denotes the stiffness of the system. $\Delta q_{e}$ and $\Delta {\dot{q}}_{e}$, respectively, represent the current elbow joint position error and current elbow joint position error. They are derived as follows [42]:

(2) $$\begin{array}{c} \Delta q_{e}=q_{h}-q_{e}\\ \Delta {\dot{q}}_{e}={\dot{q}}_{h}-{\dot{q}}_{e} \end{array}$$

where $q_{h}$ indicates the estimated human elbow joint position and $q_{e}$ represents the exoskeleton elbow joint position; ${\dot{q}}_{h}$ means the estimated human elbow joint velocity; and ${\dot{q}}_{e}$ indicates the exoskeleton elbow joint velocity. These data can be transmitted to the position controller to achieve motion control.

**Figure 2:**
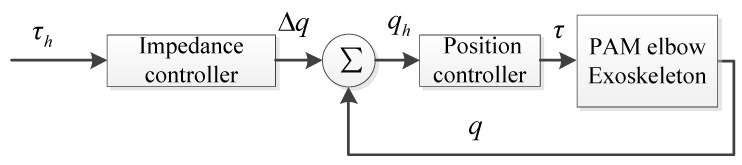
Two-layer architecture control structure: impedance control method (higher layer) and position control (lower layer).

The above control method can be designed with the traditional two-layer architecture control structure, as presented in Figure 2. In this structure, the traditional impedance controller serves as the higher layer, capturing human intention through the human–exoskeleton interactive torque signal. Moreover, it also translates the signal into a desired position value for the elbow joint. The position controller, as the lower layer, subsequently tracks the desired value with high accuracy.

To select suitable impedance parameters, the variable impedance control method based on fuzzy rules was created. It can process human–robot interaction torque information and joint position information through fuzzification to facilitate the adaptive adjustment of impedance parameters using fuzzy reasoning and defuzzification processes. This method involves the construction of fuzzy sets, the formulation of fuzzy rules, the implementation of fuzzy reasoning mechanisms, and the selection of defuzzification strategies. The fuzzy rules are generally established by experts who leverage their knowledge and experimental data to simulate the decision-making process of human experts in cases of uncertainty and ambiguity.

**Figure 3:**
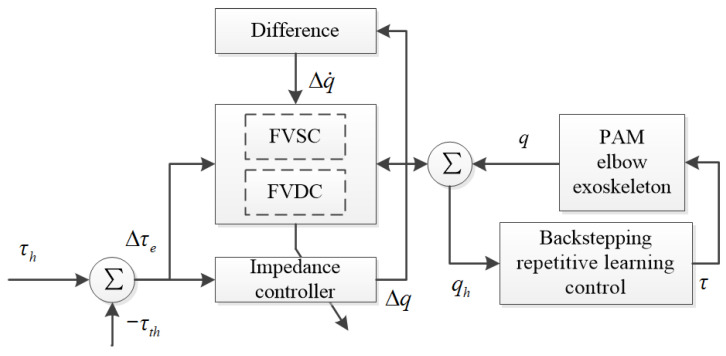
Two-layer architecture control structure: variable impedance control method based on fuzzy rules (higher layer) with backstepping repetitive learning control (lower layer).

Based on the above background, we propose a variable impedance control method (Figure 3) based on fuzzy rules, which adjusts the impedance model coefficients online using the Mamdani fuzzy algorithm. In this method, the fuzzy variable stiffness controller (FVSC) and the fuzzy variable damping controller (FVDC) are employed to adjust the stiffness coefficient and the damping coefficient of the impedance model, respectively, which improves the adaptability of the traditional impedance controller. The input of the FVSC is the current elbow joint position error (i.e., $\Delta q_{e}$) and the difference in the human–exoskeleton interactive torque (i.e., $\Delta \tau_{e}$), while the output is the stiffness coefficient correction term (i.e., ΔK). The corresponding input for the FVDC is the current elbow joint velocity error (i.e., $\Delta {\dot{q}}_{e}$) and the difference in the human–robot interaction torque, while the output is the damping coefficient correction term ΔD).

Moreover, to account for the avoidance of invalid exoskeleton motion caused by subconscious human movements (such as shaking hands and fatigue-induced arm muscle contractions and arm cramps), $\Delta \tau_{e}$ can be taken as

(3) $$\Delta \tau_{e}=\tau_{h}-\tau_{th}$$

where $\tau_{th}$ denotes the torque threshold, which determines the trigger value as a safety threshold for the motion.

**Figure 4:**
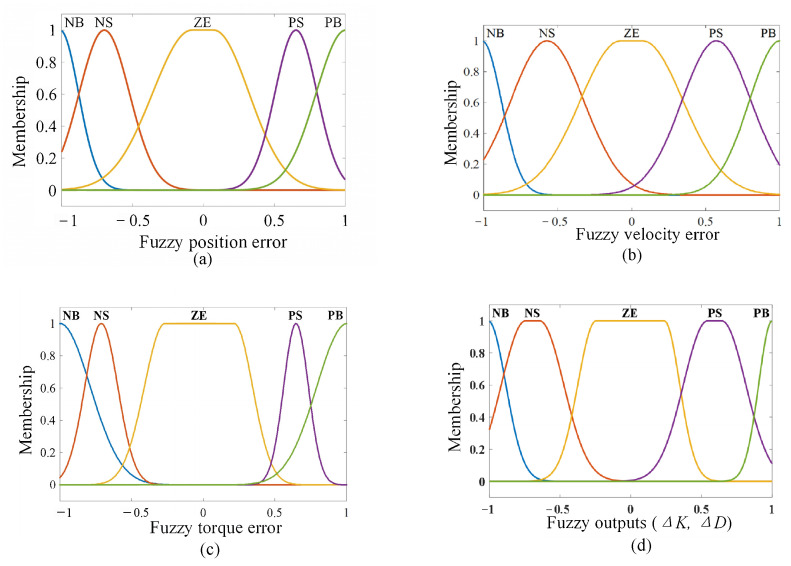
Membership function. (**a**) Δq, (**b**) $\Delta \dot{q}$, (**c**) $\Delta \tau_{e}$, and (**d**) ΔK, ΔD.

The efficiency of the variable impedance control method based on fuzzy rules is highly dependent on the construction of the FVSC and FVDC of the system. To fuzzify the inputs and outputs of the system, $\Delta \tau_{e}$ (i.e., fuzzy torque error), Δq (i.e., fuzzy position error), $\Delta \dot{q}$(i.e., fuzzy velocity error), and ΔK and ΔD (i.e., fuzzy output values) are domains in the sets of $\left[\Delta \tau_{e\min},\Delta \tau_{e\max}\right]$, $\left[\Delta q_{\min},\Delta q_{\max}\right]$, $\left[\Delta {\dot{q}}_{\min},\Delta {\dot{q}}_{\max}\right]$, $\left[\Delta k_{\min},\Delta k_{\max}\right]$, and $\left[\Delta b_{\min},\Delta b_{\max}\right]$, respectively. The normalized values of $\Delta \tau_{e}$, Δq, $\Delta \dot{q}$, ΔK, and ΔD are defined in the range of −1 to +1, where −1 presents the minimum and +1 denotes the maximum. The fuzzy sets for the above inputs and outputs are defined as negative big (i.e., NB), negative small (i.e., NS), zero (i.e., ZE), positive small (i.e., PS), and positive big (i.e., PB). The fuzzy rules were formulated with reference to quantitative knowledge, experience obtained in previous studies, and the designed membership functions are shown in Figure 4 [36,43]. The rules guiding the mapping of inputs’ and outputs’ membership functions were derived from the IF-THEN logic in Table 1. The values of the membership functions were extracted from a previous study.

For example, the control rules for the FVSC are expressed as

(4) $$If\;\Delta q\;is\;NB,\;and\;\Delta \tau_{e}\;is\;NB,\;then\;\Delta K\;is\;NB$$

Similarly, the control rules for the FVSD are expressed as

(5) $$If\;\Delta \dot{q}\;is\;NB,\;and\;\Delta \tau_{e}\;is\;NB,\;then\;\Delta D\;is\;NB$$

### 2.3. The Lower-Layer Control Method: Backstepping Repetitive Learning Control

Considering that the exoskeleton was attached to the human arm using straps, it formed an integrated system with the arm during this study. As shown in Figure 1, the ideal dynamic model of the PAM elbow exoskeleton is illustrated as two rigid links with one rotary joint. In this study, we assumed that the human’s upper-arm essentially remained stationary during rehabilitation, and the model can be simplified as a 1-DOF rigid link model containing nonlinear terms, described as

(6) $$M_{s}\ddot{q}+C_{s}\dot{q}+G_{s}+\tau_{d}+\tau_{F}=\tau +\tau_{h}$$

where $\tau_{d}$ means the disturbance torques due to the human’s subconscious movements and $\tau_{F}$ expresses the sum of the joint dynamic friction torque and uncertain torque. τ indicates the robot-assisted torque, generated from the exoskeleton.

We set $\Delta =\tau_{h}-\tau_{d}+F$, and the above system can also be expressed as follows:

(7) $$\ddot{q}=M_{s}^{-1}\tau -M_{s}^{-1}\left(C_{s}\dot{\theta }+G_{s}\right)+M_{s}^{-1}\Delta$$

While designing the lower layer of a controller, the elbow joint position of the exoskeleton should be modeled to match the human intention as the desired value calculated from the variable impedance control method based on fuzzy rules. In this study, the backstepping control method was proposed to decrease the tracking error in this nonlinear system. This method could break the complex nonlinear system into several simpler segments, allowing the methodical construction of a control strategy that enhanced stability.

In the backstepping control method, the joint position (i.e., *q*) and joint velocity (i.e., $\dot{q}$) were defined as the state variables in the PAM elbow exoskeleton system. Hence, the above equation could be rewritten as

(8) $$\begin{array}{c} {\dot{x}}_{1}=x_{2}=\dot{q}\\ {\dot{x}}_{2}={M_{S}}^{-1}\left(\tau -\left(C_{S}\dot{q}+G_{S}\right)+\Delta \right) \end{array}$$

The first-order trajectory error was defined as $e_{1}=q-q_{d}=x_{1}-x_{d}$; then, the first-order derivative of the error was ${\dot{e}}_{1}={\dot{x}}_{1}-{\dot{x}}_{1d}$. However, since ${\dot{e}}_{1}$ in real-time control tasks may cause sensitive noises, a virtual variable was defined as $\gamma ={\dot{x}}_{1}-e_{2}$. By bringing it into ${\dot{e}}_{1}$, the second-order trajectory error and its derivative could be calculated as

(9) $$\begin{array}{c} e_{2}={\dot{x}}_{1}-\gamma \\ {\dot{e}}_{2}={\ddot{x}}_{1}-\dot{\gamma }={\dot{x}}_{2}-\dot{\gamma } \end{array}$$

The virtual variable γ was expressed as ${\dot{x}}_{1d}-k_{1}e_{1}$ (where $k_{1}>0$), and the Lyapunov function (i.e., $V_{1}$) could be defined as follows:

(10) $$V_{1}=\frac{1}{2}e_{1}^{T}e_{1}+\frac{1}{2}e_{2}^{T}M_{S}e_{2}$$

By bringing γ into $V_{1}$, the derivative of $V_{1}$ was expressed as follows:

(11) $${\dot{V}}_{1}=-e_{1}^{T}k_{1}e_{1}+e_{1}^{T}e_{2}+e_{2}^{T}\left(\tau -(C_{S}\dot{\theta }+G_{S})+\Delta \right)-e_{2}^{T}M_{S}\dot{\gamma }$$

To make sure of the stability of the exoskeleton system in the dynamic form, the control law (i.e., τ) was derived as follows:

(12) $$\tau =-\Delta +C_{S}\dot{\theta }+G_{S}+M_{S}\dot{\gamma }-e_{1}-k_{2}e_{2}$$

where $k_{2}>0$ denoted the gain coefficient. By bringing Equation (12) into Equation (11), ${\dot{V}}_{1}$ could be integrated as $-(e_{1}^{T}k_{1}e_{1}+e_{2}^{T}k_{2}e_{2})$, representing the negative definite. However, the above control law could not be used directly because of the following two reasons:

(1) In the 1-DOF rigid linkage model, several parameters (e.g., $M_{s}$, $C_{s}$, and $G_{s}$) could not be determined because of the nonlinear disturbance introduced by the human in the loop.
(2) The uncertain time-varying term (i.e., Δ) could not be estimated or identified directly.

To address these problems, the control law presented in (12) was rewritten with some estimation parameters as follows:

(13) $$\tau =\tau_{1}+\tau_{2}+\tau_{3}$$

where ${\hat{M}}_{S}$, ${\hat{C}}_{S}$, and ${\hat{G}}_{S}$ denoted the initial estimation of $M_{S}$, $C_{S}$, and $G_{S}$. $\tau_{1}={\hat{C}}_{S}\dot{\theta }+{\hat{G}}_{S}+{\hat{M}}_{S}\dot{\gamma }-e_{1}-k_{2}e_{2}$. $\tau_{2}$ was used to match the estimation error caused by ${\hat{M}}_{S}$,${\hat{C}}_{S}$ and ${\hat{G}}_{S}$. $\tau_{3}$ was used to match Δ to maintain the stability of the system.

Next, we set $\tau_{2}=\left(C_{S}-{\hat{C}}_{S}\right)\dot{\theta }+\left(G_{S}-{\hat{G}}_{S}\right)+\left(M_{S}-{\hat{M}}_{S}\right)\dot{\gamma }$. Another Lyapunov candidate function was designed as

(14) $$V_{2}=V_{1}+\frac{1}{2}{\tau_{2}}^{T}k_{3}\tau_{2}$$

where $k_{3}>0$.

The derivative of $V_{2}$ was expressed as follows:

(15) $${\dot{V}}_{2}=-e_{1}^{T}k_{1}e_{1}-e_{2}^{T}k_{2}e_{2}+e_{2}^{T}\Delta +\Delta^{T}\left(e_{2}+k_{3}{\dot{\tau }}_{2}\right)$$

After denoting the adaptive law of ${\dot{\tau }}_{2}=-k_{3}^{-1}e_{2}$ and returning it into Equation (15), then ${\dot{V}}_{2}=-e_{1}^{T}k_{1}e_{1}-e_{2}^{T}k_{2}e_{2}+e_{2}^{T}\Delta$.

Moreover, the uncertain term of Δ was bounded and periodic to account for the repetitive movements in rehabilitation [25]. Following the approximate theory, it could be solved by integrating the P-type repetitive learning control (i.e., P-type RLC) scheme [23]. The following lemma was introduced to facilitate the controller design.

**Lemma 1.**  *Consider the nonlinear functions of $P\left(t\right),\tilde{P}\left(t\right)$, and $\hat{P}\left(t\right)$, and define g as belonging to the real number field. Then, there exists a relationship shown as follows:*

(16) $$\begin{array}{c} P\left(t\right)=P\left(t-T\right)\\ \hat{P}\left(t\right)=\hat{P}\left(t-T\right)+g\left(t\right)\\ \tilde{P}\left(t\right)=P\left(t\right)-\hat{P}\left(t\right) \end{array}$$

*where T>0 denotes the learning period of RLC. The upper right-hand derivative of $\int_{t-T}^{t}\tilde{P}{\left(\nu \right)}^{T}\tilde{P}\left(\nu \right)d\nu$ equals $-2\tilde{P}{\left(t\right)}^{T}g\left(t\right)-g{\left(t\right)}^{T}g\left(t\right)$. The above lemma has been previously verified.*

Using the P-type repetitive learning scheme, the control law parameter of $\tau_{3}$ was added as follows:

(17) $$\begin{array}{c} \tau_{3}={\hat{\tau }}_{3}+{\tilde{\tau }}_{3}\\ {\hat{\tau }}_{3}\left(t\right)={\hat{\tau }}_{3}\left(t-T\right)+k_{4}e_{2}t>0\\ {\hat{\tau }}_{3}\left(t\right)=0\forall t\in \left[-T,0\right] \end{array}$$

where $k_{4}>0$.

The final Lyapunov function was defined and set as $V=V_{2}+V_{3}$. We set $V_{3}$ as follows:

(18) $$V_{3}=\frac{1}{2k_{4}}\int_{t-T}^{t}{{\tilde{\tau }}_{3}}^{T}\left(\varsigma \right){\tilde{\tau }}_{3}\left(\varsigma \right)d\varsigma$$

To analyze the stability of the control system, the derivative of $V_{3}$ was expressed as follows:

(19) $${\dot{V}}_{3}\leq \frac{1}{2k_{4}}\left({\tilde{\tau }}_{3}^{T}\left(t\right){\tilde{\tau }}_{3}\left(t\right)-{\tilde{\tau }}_{3}^{T}\left(t-T\right){\tilde{\tau }}_{3}\left(t-T\right)\right)$$

Based on the above Lemma, the equation was simplified as follows:

(20) $${\dot{V}}_{3}\leq \frac{1}{2k_{4}}\left(-{\tilde{\tau }}_{3}^{T}2k_{4}e_{2}-{e_{2}}^{T}k_{4}^{2}e_{2}\right)=-{\tilde{\tau }}_{3}^{T}e_{2}-\frac{1}{2}{e_{2}}^{T}k_{4}e_{2}$$

${\dot{V}}_{3}$ and ${\tilde{\tau }}_{3}$ were introduced into $\dot{V}$, and the inequation was simplified as follows:

(21) $$\dot{V}=-e_{1}^{T}k_{1}e_{1}-e_{2}^{T}k_{2}e_{2}-\frac{1}{2}{e_{2}}^{T}k_{4}e_{2}$$

**Figure 5:**
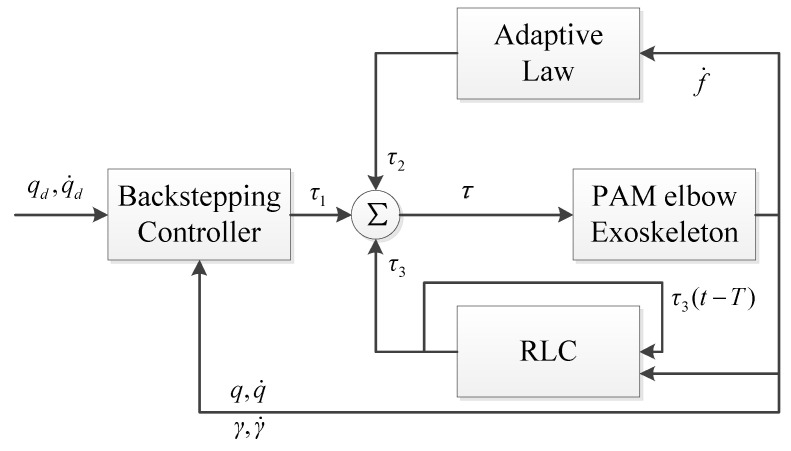
Backstepping repetitive learning control (lower layer).

Since $k_{1}$ to $k_{4}$ are all positive, $\dot{V}$ is negatively definite. Finally, the lower-layer controller is illustrated in Figure 5.

## 3. Results and Discussion

### 3.1. Simulation

To further explain the tracking performance of the purposed backstepping position loop, simulations were designed. First of all, the control plant for the exoskeleton was set as a 1-DOF linkage model because the proposed exoskeleton only had one degree of freedom at the elbow joint, as shown in Figure 1. The idealized dynamic parameters referenced the above PAM elbow exoskeleton. The main parameters of the controller are given in Table 2. Then, we assumed that the 1-DOF linkage model took a strong nonlinear disturbance, set as $5sign\left(q\right)+2sign\left(\dot{q}\right)$) Nm. In addition, the desired value of the joint position trajectory was set as

(22) $$q_{d}=\frac{\pi }{6}sin\left(\pi t\right)+\frac{\pi }{6}$$

which could mimic the passive rehabilitation training examination on the human elbow joint.

The parameter tuning process was as follows: First of all, ${\hat{M}}_{S}$, ${\hat{C}}_{S}$, and ${\hat{G}}_{S}$ were selected as the estimation values (by using the parameter identification method in the experiment) of $M_{s}$, $C_{s}$, and $G_{s}$, respectively. In the simulation, $M_{s}$ was set as 0.12, $C_{s}$ was set as 0.02, and $G_{s}$ was set as 1.41sin(q). Then, $k_{1}$ and $k_{2}$, which were the main feedback gains for $e_{1}$ and $e_{2}$, needed to be tuned. This tuning process was similar to the PD control method with dynamic model compensation. Finally, $k_{3}$ and $k_{4}$, serving as the gains for the adaptive law and RLC, respectively, were adjusted to enhance stability in the presence of nonlinear disturbances, thereby improving tracking performance. If the tracking error was still large, it was necessary to readjust $k_{1}$ and $k_{2}$ appropriately.

**Figure 6:**
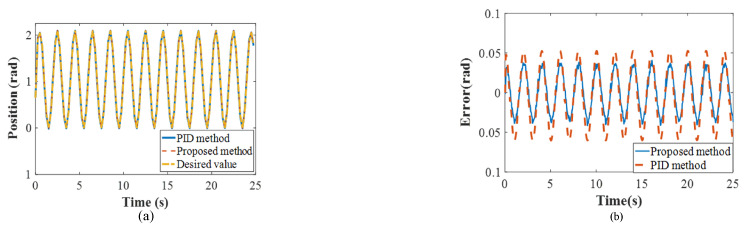
The results of the simulation ((**a**) tracking trajectory and (**b**) position error).

During the simulation, a PID controller (KP = 1500; KI = 10; and KD = 50) was selected as the comparison method, and the feedforward model of the system dynamics was compensated during the simulation to ensure fairness. Available evidence has demonstrated that the PID controller provides effective motion control for exoskeleton robots and other engineering equipment. In Figure 6, the dashed line represents the tracking trajectory and errors of the proposed controller whereas the solid line denotes the same indexes under the PID controller. The results show that both methods could successfully track the desired position trajectory, but the comparison method yielded a smoother performance than our proposed method. This was primarily due to the continuous adjustments driven by the adaptive law and the RLC controllers. However, when using the proposed controller, the maximum absolute value (MAV) of the tracking error was 0.041 rad, which was smaller than the error observed with the PID controller (i.e., 0.062 rad). Moreover, the root mean square (RMS) errors were 0.024 rad and 0.038 rad, respectively. These indices provided further evidence that the proposed controller could effectively track the performance of the PAM elbow exoskeleton.

**Figure 7:**
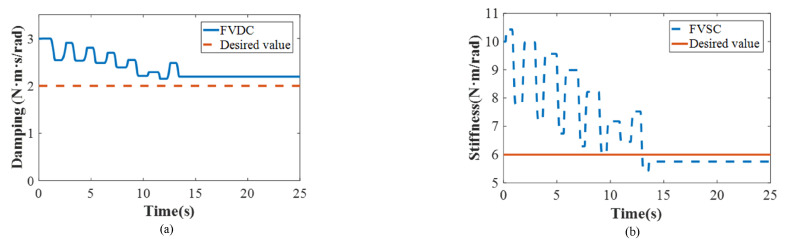
The results of the simulation ((**a**) damping and (**b**) stiffness).

We further developed another simulation to verify the interaction between the control performance above the variable impedance control method and the mimicked rehabilitation tasks. The initial assumption was that the real human–exoskeleton interactive model reflects the impedance model described in Equation (1). To develop the simulation, the impedance parameters of the human–exoskeleton system were selected as follows: *D* = 2 N·m·s/rad and *K* = 6 N·m/rad, generating the desired value of the fuzzy adaptive impedance controller. The corresponding initial impedance parameters of the proposed controller were set as folows: $D_{0}=3$ N·m·s/rad and $K_{0}=10$ N·m/rad. In the simulation, the safe threshold was set as the ideal value (0 N·m). Notably, the joint movement generated by the human followed the sine trajectory (22). It was observed that the impedance parameters of D and K converged to their desired values following several iterations by the FVSC and FVDC (i.e., 2.151 N·m·s/rad and 5.430 N·m/rad) (as shown in Figure 7), confirming the adjustment of the proposed method.

### 3.2. Experiment

**Figure 8:**
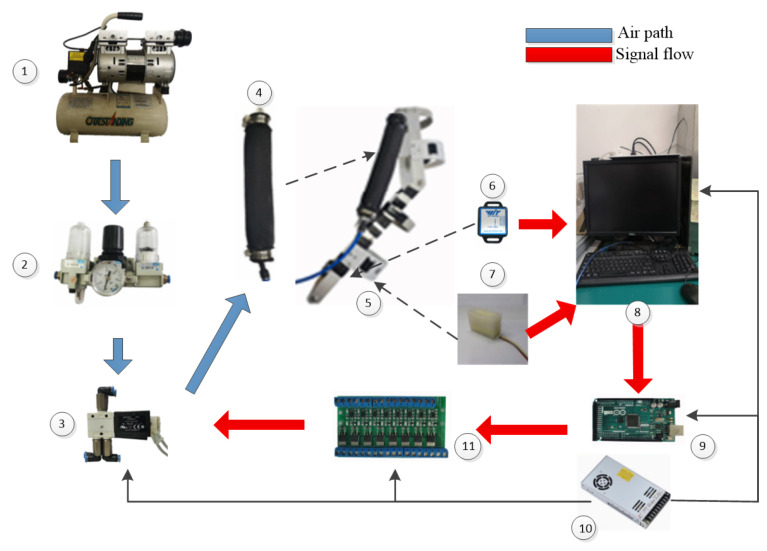
Experimental setups. (1) Air source. (2) Air filter regulator. (3) Valves. (4) PAM. (5) PAM elbow exoskeleton. (6) IMU. (7) Force sensor. (8) PC and screen. (9) Arduino mega 2560. (10) DC modular. (11) PWM modular. The black arrow indicates the power supply, and the dashed arrow indicates the implement.

In subsequent experiments, the performance of the proposed method was tested through the experiments displayed in Figure 8. As previously mentioned, the prototype of the PAM elbow exoskeleton was constructed using two PAMs as actuators, a 1-DOF linkage system as the mechanical structure, and several sensors. It performed repeated elbow flexion/extension movements to mimic the passive rehabilitation training process following the fluctuation status of the inflation and deflation of the two PAMs by controlling the two valves. The PWM signal derived from the PC was directed to the two valves and translated from the Arduino board and the PWM modular. For the sensor system, two IMUs and the soft skin sensors installed on its mechanical structure were applied for the measurement of the interaction torque and the joint position of the exoskeleton. The proposed control method was programmed in the PC using Simulink programs (Matlab 2016b, MathWorks Inc., Natick, MA, USA) based on the real-time module. The flow of the signal is marked with the red arrows and the air path is marked with the blue arrows. Comprehensive data regarding the measurement principles are described in previous studies [42].

**Figure 9:**
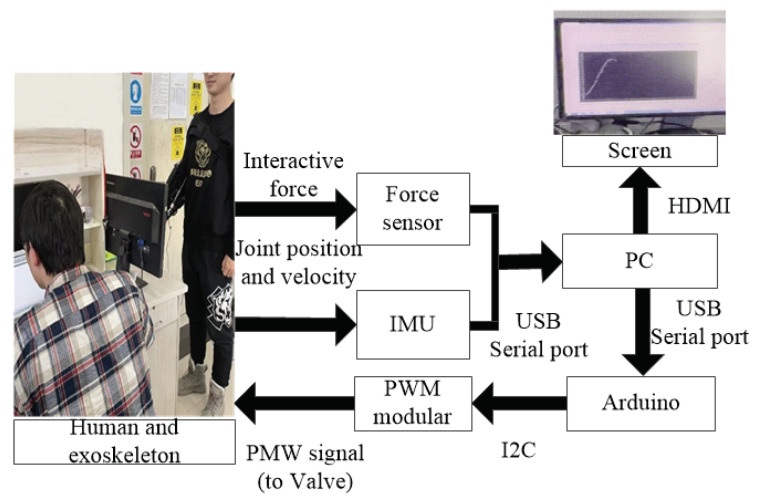
Rehabilitation experiment.

A healthy male participant (height: 181 cm, weight: 79 kg, age: 33) was enlisted to manually control the PAM elbow exoskeleton during the elbow flexion/extension rehabilitation training task, utilizing the proposed control method. The primary goal of the task was to follow a sinusoidal trajectory with a frequency of 0.1 Hz and an amplitude of 0.2 rad, which served as the reference for the joint movement. This reference trajectory was shown on the PC screen but was not embedded within the program. Elbow flexion/extension training can facilitate the reorganization of neurons and the regeneration of cells in the affected area of stroke patients, thereby significantly improving and restoring their movement abilities. During the experiment, the participant was able to produce the interactive force on the force sensors which could control the exoskeleton to raise up and put down his forearm, as shown in Figure 9. To ensure experimental safety and eliminate any ethical issues, the subject was instructed not to wear the exoskeleton instead of manipulating it under the supervision of a safety officer. Before experimentation, the participant was adequately trained and was informed about the operational requirements. In case of unexpected events, the participant was instructed to stop the operation by pressing the emergency switch. In addition, care was taken to ensure that the experimental process did not attract any ethical issues.

**Figure 10:**
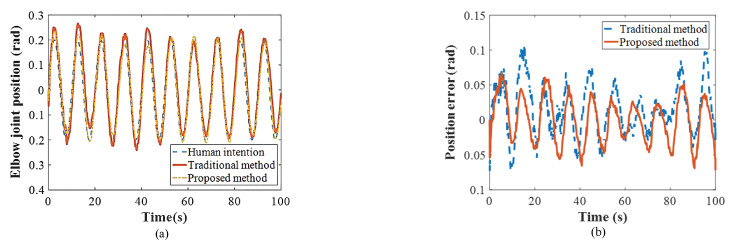
Experimental results. Parameters: (**a**) joint position; (**b**) position error (between human intention and actual position).

**Figure 11:**
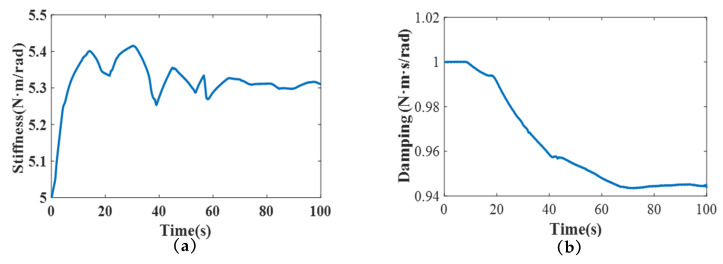
Experimental results. Parameters: (**a**) stiffness; (**b**) damping.

**Figure 12:**
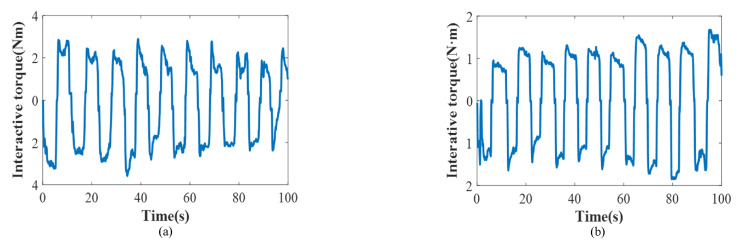
Experimental results: interactive torque (**a**) traditional method; (**b**) proposed method.

The experimental results are presented as shown in Figure 10, Figure 11 and Figure 12. First of all, a traditional impedance method (K = 5 N·m/rad; D = 1 N·m·s/rad) was used as a comparative method in the experiment. The principle of the traditional impedance method can be found in Figure 2. To verify the performance of the experiment, two targets were selected: the accuracy of the position and impedance adjustment capability. The control method was modeled to improve the tracking performance of the PAMs actuator as well as to match with human intentions to achieve high positional accuracy. Figure 10 shows that the two algorithms performed periodic active rehabilitation training tasks, owing to their similar low-level control methods. The different impedance control methods influenced human behavior, with the proposed algorithm resulting in a lower tracking position error. Specifically, the RMS error for the proposed method was 0.032 rad, and the MAV error was 0.628 rad, compared to the comparative method, which recorded errors of 0.041 rad and 0.973 rad, respectively. These findings indicate that the proposed method more effectively aligned with human motion intentions.

Meanwhile, to further prove that the proposed control algorithm has the ability to adjust impedance parameters timely, the various impedance parameters and the interactive torque were also recorded. In Figure 11, D converged from 1 to 0.944 N·m·s/rad and K converged from 5 to 5.341 N·m/rad with several iterations. From the results, the proposed algorithm simplified the impedance parameter identification process of D and K and enabled them to be adjusted by the user through fuzzy control. In addition, the convergence of impedance parameters not only signified the user’s adaptation to the exoskeleton’s operation throughout the rehabilitation process but also suggested that the exoskeleton–human system achieved greater stability. Moreover, the proposed approach exhibited a significantly lower interactive torque compared to the traditional impedance method. Specifically, the maximum absolute value of the interactive torque was smaller than 2 N·m (1.917 N·m, Figure 12), illustrating the safety of the human, similarly to a previous report [42]. In contrast, the traditional impedance control method generated a large interactive torque (i.e., the maximum value was 3.583 N·m, 46.49% less than the traditional impedance control). These results demonstrate that the proposed algorithm possessed adaptive capabilities, with its impedance parameters evolving over time to closely match the dynamics of the actual human–exoskeleton system. Moreover, the lower interactive torque achieved by the proposed controller highlighted its suitability and comfort for the task at hand. Therefore, the two-layer control method presented here shows promising potential for application in real robot-assisted rehabilitation settings.

## 4. Conclusions

This study developed a two-tiered control strategy for the PAM elbow exoskeleton for use in rehabilitation. To conclude, the FVSC and FVDC were designed to optimize virtual impedance in the higher layer of the control, while the backstepping repetitive learning control was implemented for the position control in the lower-layer.

After, stability proof, simulation, and experimental setups were designed to emulate actual rehabilitation tasks. From the results, we see that the root mean square of the tracking error was 0.032 rad and the steady-state interactive torque was 1.917 N·m. These reveal that the proposed method had a lower interactive torque and could adjust the impedance parameters, unlike the traditional impedance methods, while maintaining a good position-tracking performance.

As mentioned above, the proposed method has great potential for application in robotic-assisted rehabilitation settings. The proposed exoskeleton still relies on a rigid linkage structure; thus, future research will focus on developing soft exoskeletons and their control methods.

## Figures and Tables

**Table 1:** Fuzzy rules of FVSC and FVSD.

	Δτ	NB	NS	ZE	PS	PB
$\Delta \dot{q},\Delta q$						
NB		NB	NS	NS	PS	PS
NS		NS	NS	ZE	ZE	PS
ZE		NS	ZE	ZE	ZE	PS
PS		NS	ZE	ZE	PS	PS
PB		NS	NS	PS	PS	PB

**Table 2:** The parameters of the controller.

${\hat{M}}_{S}$	${\hat{C}}_{S}$	${\hat{G}}_{S}$	$k_{1}$	$k_{2}$	$k_{3}$	$k_{4}$	*T*
0.12	0.02	1.41sin(q)	11	2	0.1	1	1

## Data Availability

Data are contained within the article.

## Abbreviations

The following abbreviations are used in this manuscript:

PAM	Pneumatic artificial muscle
FVSC	Fuzzy virtual stiffness controller
FVDC	Fuzzy virtual damping controller
RMS	Root mean square
